# Phytoplankton size-diversity mediates an emergent trade-off in ecosystem functioning for rare versus frequent disturbances

**DOI:** 10.1038/srep34170

**Published:** 2016-10-17

**Authors:** S. Lan Smith, Sergio M. Vallina, Agostino Merico

**Affiliations:** 1 Ecosystem Dynamics Research Group, Research Centre for Global Change, JAMSTEC, Yokohama, Japan; 2 Institute of Marine Sciences (CSIC), 08003 Barcelona, Catalonia, Spain; 3 Systems Ecology, Leibniz Center for Tropical Marine Ecology (ZMT), Bremen, Germany; 4 Physics & Earth Sciences, Jacobs University, Bremen, Germany

## Abstract

Biodiversity is known to be an important determinant of ecosystem-level functions and processes. Although theories have been proposed to explain the generally positive relationship between, for example, biodiversity and productivity, it remains unclear which mechanisms underlie the observed variations in Biodiversity-Ecosystem Function (BEF) relationships. Using a continuous trait-distribution model for a phytoplankton community of gleaners competing with opportunists, and subjecting it to differing frequencies of disturbance, we find that species selection tends to enhance temporal species complementarity, which is maximised at high disturbance frequency and intermediate functional diversity. This leads to the emergence of a trade-off whereby increasing diversity tends to enhance short-term adaptive capacity under frequent disturbance while diminishing long-term productivity under infrequent disturbance. BEF relationships therefore depend on both disturbance frequency and the timescale of observation.

Biodiversity is an important determinant of ecosystem function, including productivity, which impacts the extent to which ecosystems can provide many resources and services valued by humans^1^,^2^. Intensive observations during the past two decades have revealed a generally positive, and in some cases unimodal^3^, relationship between the level of diversity in a community or ecosystem and measures of its function, such as productivity. However, the considerable variability observed in such Biodiversity-Ecosystem Function (BEF) relationships remains largely unexplained^1^,^2^,^4^,^5^. Uncertainty remains about the ecological mechanisms responsible for the enhancement of productivity with increasing diversity, particularly the relative contributions of species complementarity (i.e., niche partitioning such that different species are better able to exploit resources under different conditions) versus selection for the fittest species^2^,^6^,^7^,^8^.

Plankton are often taken as ideal model organisms for both empirical^9^ and modelling^10^ studies in ecology, in part because of the fast generation times and great numbers typical of plankton species. In addition, phytoplankton are important because they constitute the base of the food chain in aquatic environments. Trait-based approaches are being applied successfully in empirical studies of BEF for microbes and plankton^11^, as well as in many recent modelling studies of plankton ecosystems and BEF relationships^3^,^12^,^13^,^14^. Recent studies^12^,^15^,^16^ have modeled size-structured plankton ecosystems based on reported allometries for phytoplankton trait values^17^,^18^. However, regardless of whether such models explicitly represent many different idealised species^19^ or assume continuous trait distributions^20^,^21^,^22^, modeled biodiversity tends to decrease unrealistically over time as a result of competitive exclusion^3^,^23^.

Here we examine diversity-productivity relationships with the aim of clarifying their underlying mechanisms, using a trait-based plankton ecosystem model with size as the master trait for phytoplankton. Simplified size-scalings^13^,^24^ of Monod growth parameters are used to relate two key functional traits, nutrient affinity and maximum growth rate, via a gleaner-opportunist trade-off. This and similar trade-offs are important determinants of competitive outcomes in many ecosystems^25^,^26^,^27^. We compare the dynamic response of the phytoplankton community, and of an implicitly represented community of zooplankton grazing on them, to pulsed perturbations of varying frequency and intensity. The perturbations are imposed as non-selective mortality events^5^,^28^, each killing off some fraction of the phytoplankton independent of their size, because this allows controlled tests of the resilience of communities having different size distributions. In order to investigate the combined effects of biodiversity and disturbance frequency on diversity-productivity relationships, the perturbations are imposed on communities having different levels of size diversity.

We compare the results obtained using two recently developed formulations for sustaining diversity, each having one adjustable parameter allowing different levels of diversity to be maintained, along with a control model in which diversity is not sustained, i.e. decreases steadily with time. The Trait Diffusion (TD) model was recently developed^29^ as a means of representing the maintenance of diversity via endogenous (to the phytoplankton population or community) mechanisms that cause traits to vary through subsequent generations. These mechanisms could include mutation, which alters the genotype, as well as trans-generational phenotypic plasticity, i.e., independent of changes in the offspring genotype. Alternatively, an exogenous mechanism is the recently developed ‘Kill-the-Winner’ (KTW) formulation for grazing^30^, which has been shown to enhance biodiversity through predator-mediated coexistence in a model that discretely resolves many different species^3^. Our aim is to understand how these two different approaches to sustaining diversity, and different levels of functional diversity, impact BEF relationships under different frequencies of disturbance (perturbation).

The model applied (Fig. 1) is in essence a typical Nutrient- Phytoplankton-Zooplankton- Detritus (NPZD) model^31^, with two important modifications: (1) a continuous (log-normal) size-distribtution with size-scaling of traits for phytoplankton^13^,^16^ together with the ‘adaptive dynamics’ equations^20^,^21^,^22^ for the community size distribution, and (2) the TD and KTW formulations for sustaining phytoplankton diversity. Different levels of phytoplankton size diversity are sustained by adjusting a single parameter for either the TD or KTW formulation, respectively. For each level of diversity, the model is first allowed to equilibrate for a spin-up period (90 d) during which it simulates a batch incubation, and then the periodic disturbances are simulated by periodic nutrient additions as in semi-continuous batch cultures^28^,^32^. See the Methods for details.

## Results

### Selection effect

**Figure 2:**
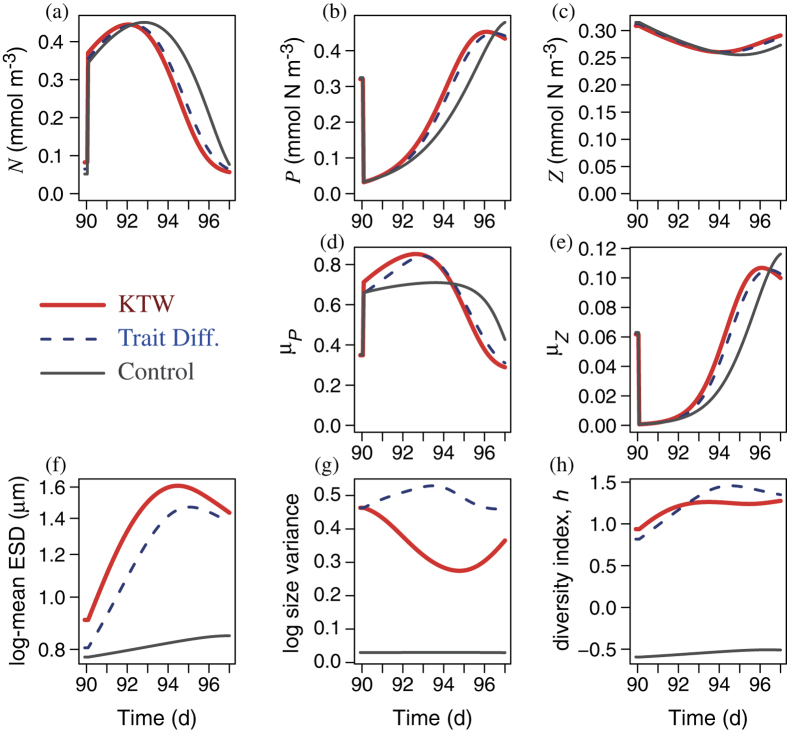
Short-term (7 d) response to a single instantaneous, non-selective disturbance event (90% phytoplankton mortality), applied after a 90 d equilibration (spin-up) period. Standing stocks of (**a**) nutrient, (**b**) phytoplankton, and (**c**) zooplankton biomass, specific growth rates of (**d**) the phytoplankton community and (**e**) zooplankton, (**f**) log mean size (as Equivalent Spherical Diameter, ESD), (**g**) log size variance, and (**h**) continuous size diversity, *h*. The KTW formulation was applied with its default value of *α* = 2, and the TD parameter was adjusted to a value of *ν* = 0.04048 in order to give the same log size variance at the end of the spin-up period.

**Figure 3:**
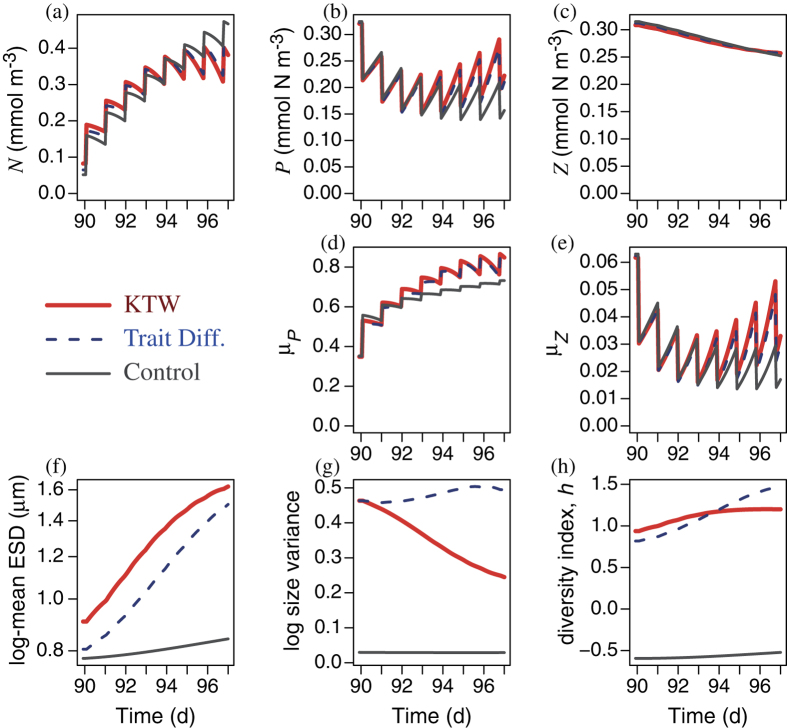
Short-term (7 d) response to a series of 7 daily non-selective mortality events, applied after a 90 d equilibration (spin-up) period. In each event 33% of the phytoplankton community was instantaneously killed off. Panels and symbols are as defined in Fig. 2.

In the short-term (7 d), more diverse phytoplankton communities respond and recover faster after a single high intensity disturbance, mimicking a catastrophic kill-off event (Fig. 2). More diverse communities also recover faster under a series of less intense disturbances (once per day), mimicking a less catastrophic but persistent stressor (Fig. 3). With more diverse communities the mean size shifts faster (via species sorting) in the direction that tends to increase growth rate, as seen for the KTW and TD models in Figs 2f and 3f. This reflects rapid selection^6^ in favor of the size having the optimal strategy subject to the assumed trade-off. Equation S-1 (Supplementary online Methods) represents this mathematically. This is the reason that the more diverse communities (in both KTW and TD cases) are able to take up the newly available nutrient faster, and hence recover more quickly from the disturbance, compared to the control model. The selection effect thus yields a more resilient response, which enhances the short-term Adaptive Capacity (AC) for sufficiently diverse communities.

After the onset of disturbances, the nutrient concentration increases (Figs 2a and 3a), and phytoplankton initially decrease and then recover somewhat (Figs 2b and 3b). Zooplankton gradually decline (Figs 2c and 3c) because of the reduced availability of prey. This trophic cascade has differential impacts on size diversity with the two diversity-sustaining mechanisms considered.

With the KTW formulation^30^ the foraging effort on any given prey size is proportional to the fraction of prey biomass having that size, raised to the power *α* (equation S-10, Supplementary online Methods). Hence with *α* = 1, as in the control model, prey-specific foraging effort is independent of the relative abundance of prey (equation S-11), and therefore grazing does not alter prey diversity. For *α* > 1 foraging effort is concentrated on the more abundant size classes, and more so with increasing *α*, so that grazing flattens the prey size distribution, which sustains phytoplankton diversity. After the onset of disturbances, the reduction in zooplankton biomass reduces the grazing pressure on phytoplankton and hence the effectiveness of the KTW formulation. Hence the size variance tends to decrease with the KTW formulation (Figs 2g and 3g). The diversity index, *h*, nevertheless tends to increase because it depends on both size variance and log-mean size (equation 6), the latter of which increases (Figs 2f and 3f) in response to the elevated nutrient concentrations.

With the TD formulation^29^, the rate of trait diffusion, *ν*, is the probability that the next generation of phytoplankton will differ in size (and hence functional trait values) from the current generation. Hence, for *ν* = 0, as in the control model, growth does not enhance diversity, and for any positive value of *ν*, growth enhances phytoplankton size diversity. During the 7 d period after the onset of disturbances, the elevated nutrient concentrations enhance phytoplankton growth rate, and thereby enhance the effectiveness of the TD formulation. This is why the TD formulation produces greater size variance (Figs 2g and 3g) and diversity index (Figs 2h and 3h), compared to the KTW formulation.

### Disturbance frequency

**Figure 4:**
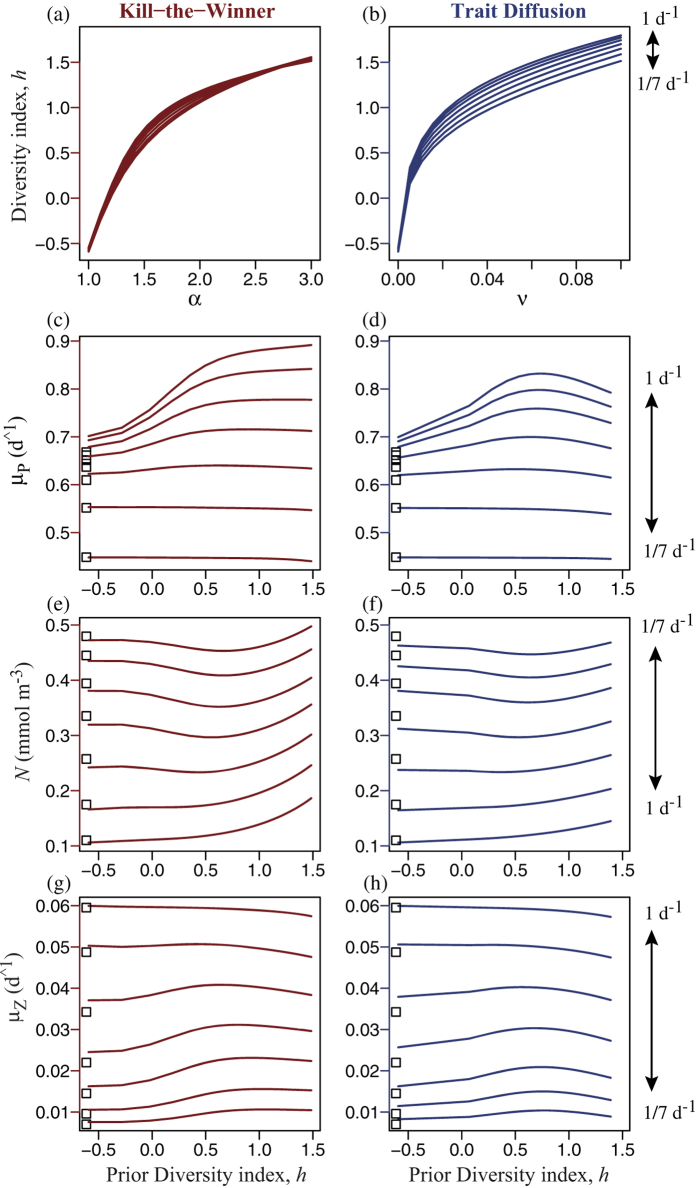
Size diversity index, *h*, averaged over 7 d following the first disturbance versus the (**a**) KTW parameter *α*, and (**b**) TD parameter *ν*. Vertical arrows specify frequencies of disturbance. Short-term Adaptive Capacity (AC) is quantified by avg. values over the same 7 d of: mean specific growth rate, *μ*_*P*_, for the phytoplankton community (**c**,**d**), nutrient concentration, *N* (**e**,**f**), and specific growth rate of zooplankton, *μ*_*Z*_ (**g**,**h**), each plotted vs. the size diversity index before the first disturbance. White squares (left) show the corresponding values for the single size having the fastest avg. specific growth rate at each frequency.

With respect to short-term AC (7 d average), the diversity-productivity relationship depends on both the frequency of disturbance and the level of size diversity in the community prior to disturbance (Fig. 4). Here we examined a range of disturbance frequency from once per day (the same time scale as the maximum growth rate for phytoplankton, Table S1, Supplementary online Methods) to once per week (the timescale for the short-term average considered). For each pulse frequency, the level of phytoplankton size diversity was controlled by varying either the KTW parameter, *α*, (Fig. 4a) or the rate of trait diffusion, *ν* (Fig. 4b). More diverse phytoplankton communities tend to be more productive (greater specific growth rate) at disturbance frequencies greater than once per 5 d (Fig. 4c,d), except that with the TD formulation productivity declines for the highest levels of prior diversity, giving a unimodal pattern (Fig. 4d). However, under less frequent disturbance, more diverse phytoplankton communities are slightly less productive with both formulations. More productive communities draw nutrients down to lower concentrations, giving an inverted pattern (Fig. 4e,f). In contrast to phytoplankton, zooplankton productivity decreases with increasing phytoplankton diversity under frequent disturbance, but has a unimodal pattern (greatest at intermediate diversity) under infrequent disturbance (Fig. 4g,h), with both KTW and TD.

**Figure 5:**
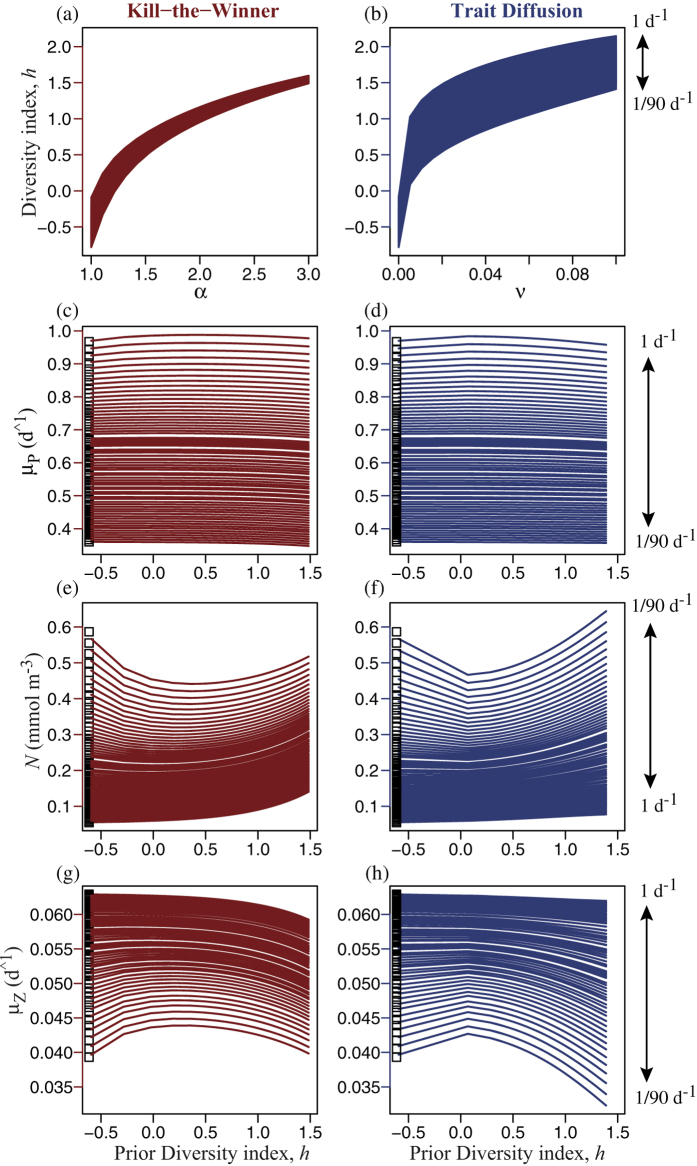
Size diversity index, *h*, averaged over 90 d following the first disturbance versus the (**a**) KTW parameter *α*, and (**b**) TD parameter *ν*. Vertical arrows specify frequencies of disturbance. Long-term Productivity (LP) is quantified by avg. values over the same 90 d of: mean specific growth rate, *μ*_*P*_, for the phytoplankton community (**c**,**d**), nutrient concentration, *N* (**e**,**f**), and specific growth rate of zooplankton, *μ*_*Z*_ (**g**,**h**), each plotted vs. the size diversity index before the first disturbance. White squares (left) show the corresponding values for the single size having the fastest avg. specific growth rate at each frequency.

The level of diversity also impacts Long-term Productivity (LP), averaged over 90 d (Fig. 5). Here we examined a range of disturbance frequency from once per day to once per 3 months (the timescale of long-term average considered, which was chosen to match that of seasonal variation). For low frequencies of disturbance, LP of phytoplankton decreases slightly with increasing diversity (Fig. 5c,d). Under more frequent disturbance, it is maximal at intermediate levels of diversity, with either diversity-sustaining mechanism. The patterns for nutrient and LP for zooplankton are similar to those in the short-term case (Fig. 4).

Increasing diversity from low to intermediate levels tends to enhance AC (Fig. 4c,d), and to a lesser degree LP (Fig. 5c,d), of phytoplankton under frequent disturbance. However, increasing diversity also consistently diminishes both AC and LP under infrequent disturbance. This constitutes an emergent trade-off, in that more diverse phytoplankton communities are able to respond more quickly to disturbance, but grow more slowly than less diverse communities during long periods without disturbance. That is, sustaining diversity, by either endogenous or exogenous mechanisms, leads to two opposite effects: (1) a short-term resilience by allowing optimal sizes to be present and selected in the community by the rapidly changing environmental conditions, and (2) a slightly lower long-term productivity by also harboring sub-optimal sizes during periods of relatively constant conditions. Therefore, the optimal level of diversity depends on the frequency and intensity of disturbances. Also, for any given value of either the TD parameter, *ν*, or the KTW parameter, *α*, greater diversity is sustained under more frequent perturbations, both in the short-term (Fig. 4a,b) and in the long-term (Fig. 5a,b).

The level of phytoplankton diversity also affects the specific growth rate of zooplankton under different frequencies of disturbance. With the grazing equation employed in our model^30^ the total grazing rate depends only on the total prey concentration, not on the distribution of prey. Hence prey diversity can only indirectly impact trophic transfer, in that the level of phytoplankton size diversity impacts the community average growth rate of phytoplankton, and in turn the amount of prey available to zooplankton.

### Complementarity effect

**Figure 6:**
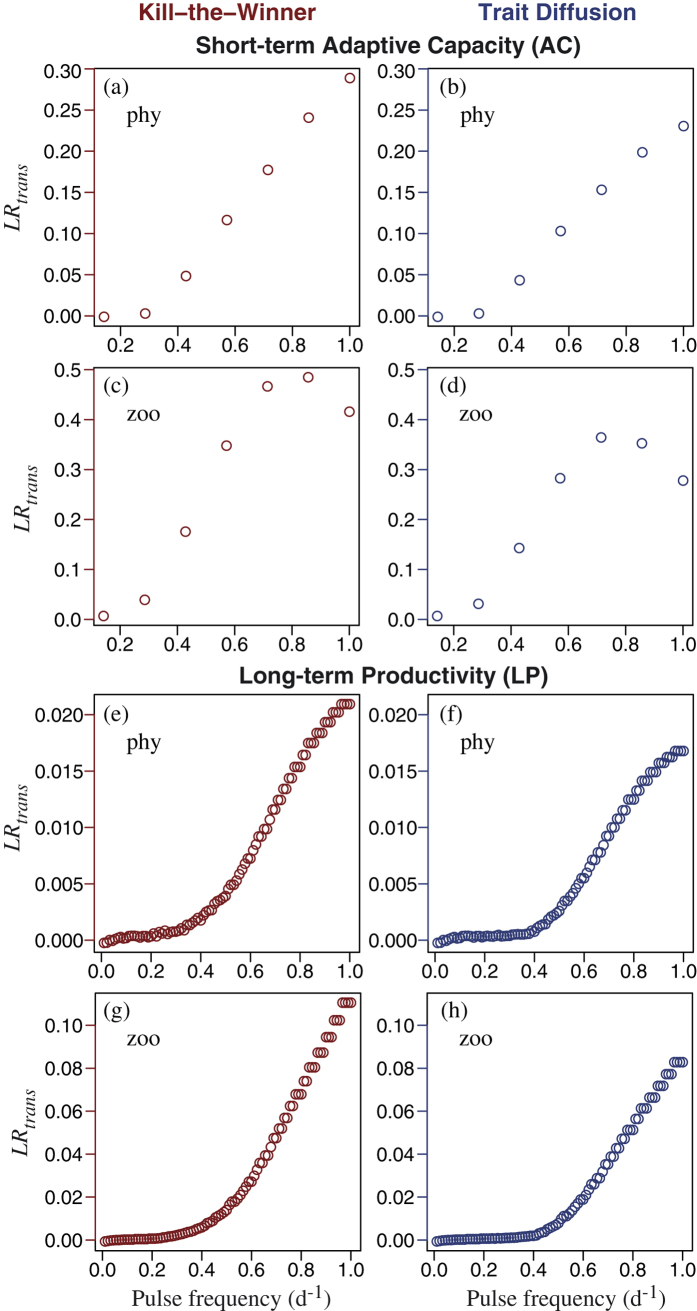
Maximal values of *LR*_*trans*_ for phytoplankton for each pulse frequency (horizontal axis) in the short-term (7 d) case as obtained using (**a**) KTW and (**b**) TD, and corresponding values for zooplankton with (**c**) KTW and (**d**) TD. The same for the long-term (90 d) case for phytoplankton: (**e**,**f**), and zooplankton: (**g**,**h**).

The degree of temporal complementarity is made clear by comparing the results of the trait-based community model to those for an otherwise identical model including only the single size class of phytoplankton having the fastest average specific growth rate over the period of interest, for each pulse frequency, respectively (white boxes shown to the left in Figs 4 and 5). At low frequencies of disturbance the single most productive size is as productive as the diverse community having optimal diversity, but under more frequent disturbance the diverse community is more productive (i.e., greater complementarity) precisely because it is able to shift its mean size to optimise growth rate. As a result, the nutrient is drawn down to lower concentrations at the optimal level of diversity. The degree of complementarity is revealed clearly by the log ratio of transgressive overyielding, *LR*_*trans*_, which takes on the value unity if the community as a whole is just as productive as its most productive member (size class or species), and takes on values greater than unity if the diverse community is more productive than its most productive member. *LR*_*trans*_ was greater for AC (Fig. 6a–d) than for LP (Fig. 6e–h), by approximately a factor of 10 for phytoplankton and a factor of 5 for zooplankton.

By contrast, complementarity enhances the average specific growth rate of zooplankton more so at intermediate frequencies of disturbance in the short-term (Fig. 6c,d). This is because at the highest frequencies of disturbance, the zooplankton do not have time to exploit the enhanced production by phytoplankton. Simulations with a faster grazing rate (4 d^−1^) yielded a monotonic increase in *LR*_*trans*_ for zooplankton with increasing disturbance frequency (not shown).

### Endogenous vs. exogenous maintenance of diversity

**Figure 7:**
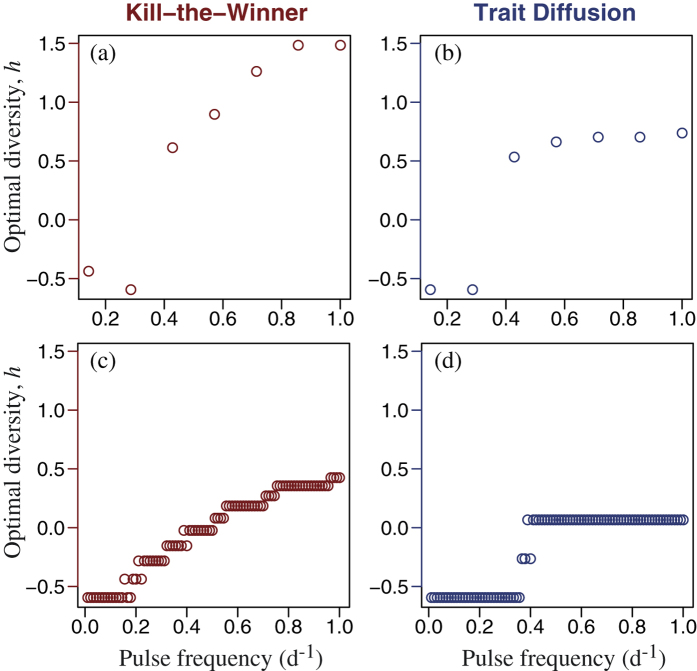
Optimal values of size diversity, *h*, for phytoplankton for each pulse frequency (horizontal axis) in the short-term (7 d) case as obtained using (**a**) KTW and (**b**) TD, and corresponding values for the long-term (90 d) case for phytoplankton: (**c**,**d**).

Although the broad patterns are similar with both KTW and TD, there are differences. Most important, the strength of the KTW effect depends on the grazing pressure, and hence on the zooplankton biomass, whereas TD is independent of the predator. With the KTW mechanism when the biomass of the predator is reduced because its prey has been diminished after intense pulse mortality events (Fig. 2c), the phytoplankton size-diversity decreases due to weaker predator-mediated coexistence. This is why after intense disturbance the TD mechanism sustains more diversity than the KTW mechanism, both in terms of size variance (Fig. 2g) and continuous entropy (Fig. 2h), when the biomass of the predator is reduced because its prey has been diminished (Fig. 2c). The same is true for scenarios of multiple pulses of lower intensity (Fig. 3). With both KTW and TD the optimal level of diversity, in terms of maximising the community average growth rate of phytoplankton, increases in a saturating fashion with the frequency of disturbance, although it saturates at lower frequencies and lower levels of diversity for TD, compared to KTW (Fig. 7). This is because high disturbance frequencies diminish the marginal effectiveness of the KTW mechanism (i.e., the slope of diversity index versus parameter *α* decreases with increasing pulse frequency in Fig. 4a and to a lesser extent in Fig. 5a). Therefore, with KTW at high values of *α*, size diversity during the period of disturbance does not become high enough to reduce the community average growth rate to the extend that it does with TD at high values of parameter *ν* (compare Fig. S1c,d for the short-term response and Fig. S2c,d for the long-term).

## Discussion

This study has demonstrated that continuous trait-distribution models using the ‘adaptive dynamics’ approach^20^,^21^,^22^ can be useful tools for studying BEF relationships. We have presented the first derivation of the KTW formulation^3^,^30^ for such continuous trait-distribution models, which we hope may prove useful for future studies.

In line with the suggestion by Krause *et al*.^11^, we argue that BEF relationships for marine plankton can be more easily and meaningfully understood by considering size diversity, or at least functional diversity with respect to specific trait values, in addition to species diversity. This is because the species diversity alone does not necessarily reveal the functional diversity of the community, which depends on the degree of niche packing, i.e., on how much the species present differ in their functional trait values^8^.

An expected trade-off has previously been described^33^ between greater productivity of relatively few species under stable conditions (for which they are optimised) versus the longer-term stability of more diverse communities, which may not be as productive during (typically shorter) periods without disturbance because they contain more sub-optimal members. Here we have clarified the roles of selection and complementarity, as mediated by size diversity and the frequency of disturbance, in producing this emergent trade-off. Specifically, greater size diversity, which implies greater functional diversity, of phytoplankton enhances the short-term Adaptive Capacity (AC) of the community when subjected to frequent or intense perturbations, at the expense of its Long-term Productivity (LP) over extended periods relatively free of disturbance.

Our model phytoplankton community exhibits both selection effect in the sense that at each given time the environment is selecting the most successful size classes, and complementarity effect in the sense that a diverse community of species can potentially exploit more efficiently over time a broad range of nutrient conditions by shifts in species compositions, as inferred by changes in the optimal mean trait. Greater size (hence functional) diversity enhances directly the selection effect (i.e. the rate of change of the mean size), which in turn enhances complementarity. Thus to the argument of Petchey^7^ that complementarity can enhance the selection effect, we add that the reverse can also occur in dynamic contexts such as considered herein. Although the characteristic time scales for plankton are necessarily shorter than those for longer-lived organisms, including many terrestrial plants, the same general mechanisms and tendencies may be expected. One reason for this is that either the same gleaner-opportunist trade-off assumed herein or similar trade-offs are known determinants of competitive outcomes in both aquatic and terrestrial ecosystems^25^,^26^ as well as in microbial ecology^27^.

Although we assumed a gleaner-opportunist trade-off in formulating our model, and indeed trade-offs are a general feature of theories and models of coexistence^2^,^26^, we emphasise that the assumed trade-off alone is insufficient to produce the emergent trade-off identified between AC and LP. This latter trade-off emerges because under frequent disturbance functional diversity enhances directly the selection effect, which in turn enhances indirectly the degree of complementarity in time^8^,^33^. Our results provide an answer to the question raised by Cardinale *et al*.^8^: ‘Why do we generally fail to find transgressive overyielding in experiments despite the evidence for complementarity among species?’ It may be relatively rare for experiments to include the particular combinations of diversity and disturbance frequency required to give rise to transgressive overyielding, which occurs when the community as a whole is more productive than even its most productive component species in isolation^8^. Our findings highlight the combined role of biotic and abiotic factors in determining overall ecosystem response^33^, as well as the combined roles of complementarity and selection.

We also found an explanation for the persistent difficulty in quantifying and understanding BEF relationships^4^,^5^,^28^. The optimal level of diversity, in terms of ecosystem function, depends on the frequency and intensity of disturbances to which an ecosystem is exposed (Fig. 7) and, as found previously^3^, on the temporal scale at which the ecosystem response is evaluated (Figs 4 and 5). Our results agree with previous findings of a diminishing marginal enhancement of resource use efficiency with increasing diversity^34^ and that process rates (i.e., ecosystem function) do not in all cases increase with increasing diversity^33^. Furthermore, we have identified how phytoplankton diversity, via enhancing primary production –i.e., ‘transgressive overyielding’^8^,^33^– can indirectly enhance trophic transfer, which may have important implications for the role of phytoplankton diversity in sustaining higher trophic levels in aquatic ecosystems.

Analysis of extensive data from oceanic observations of plankton has recently revealed that trophic transfer is hindered by prey size diversity and enhanced by predator size diversity^35^, which is a more direct and complex effect than the indirect effect of prey size diversity on trophic transfer identified herein. Finally, although the size-based gleaner-opportunist trade-off that we assume is widely observed and appropriate to this theoretical study, our simple model cannot provide a comprehensive representation of phytoplankton diversity in the ocean. Future studies using more elaborate models incorporating more detailed mechanisms will be required to test against observations and explore implications.

## Methods

### Model Equations: Size-scaled Monod growth kinetics

For the sake of simplicity, we apply the familiar Monod growth kinetics for phytoplankton, where the specific growth rate, *μ* (d^−1^) depends only on the nutrient concentration, *N* (mmol N m^−3^) as follows:


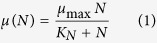


where *μ*_max_ (d^−1^) is maximum growth rate and *K*_*N*_ (mmol N m^−3^) is the half-saturation value. We define size-scalings for the model parameters^13^ in terms of *l*, the log of the equivalent spherical diameter (ESD):


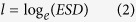


Then the size scalings of the two Monod growth parameters are:


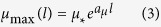



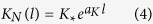


For this simple test case, we assume *a*_*μ*_ = 1 and *a*_*K*_ = 2, which gives rise to a trade-off between competitive ability at low *N* (favoring small cell size) vs. high *N* (favoring large cell size). In terms of the affinity (initial slope of growth rate with respect to *N*), *A* = *μ*_max_/*K*_*N*_, the chosen parameters specify a linear trade-off between *A* and *μ*_max_, just as in the case of Optimal Uptake kinetics^36^. In other words, the size-scaling factor for affinity, *a*_*A*_ = *a*_*μ*_ − *a*_*K*_ = −1. The size-scaled equation for specific growth rate is then:


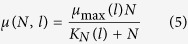


with parameter values listed in Supplementary Table S1. The specified increase in *μ*_max_ with size agrees with reported size scalings for phytoplankton smaller than approximately 5 *μ*m in diameter, but not for larger size classes^12^,^18^. We therefore confine the conditions of our simulations to those under which phytoplankton smaller than 5 *μ*m dominate.

The above equations for phytoplankton growth were implemented in a continuous size-distribution model using the ‘adaptive dynamics’ approach^20^,^21^,^22^ to represent the phytoplankton biomass, mean size, and size variance as prognostic variables (Supplementary online Methods). Zooplankton were included using the Kill-the-Winner grazing formulation^30^ here modified for a continuous size distribution of prey (Supplementary online Methods).

### Simulations

The model was set up to simulate semi-continuous batch cultures^28^,^32^ with periodic nutrient additions. With each addition of nutrient-rich water, plankton are allowed to overflow (pulsed mortality) as some fraction of the volume of the incubation vessel is displaced. Each nutrient addition constitutes a non-selective mortality event, i.e., kills off a certain fraction of the phytoplankton independent of their size, which is considered ideal for testing the resilience of the community^5^,^28^. Here we also assume that each time the nutrient added exactly balances that contained in the fraction of phytoplankton killed off, which is equivalent to assuming instantaneous regeneration of all nutrient contained in the fraction of phytoplankton lost to mortality.

Different frequencies of disturbance (i.e., nutrient addition) were applied, assuming 50% mortality of phytoplankton with each disturbance. The average response of the plankton ecosystem was quantified over 7 d (short-term), termed the short-term Adaptive Capacity (AC), and 90 d periods, termed the Long-term Productivity (LP). As measures of ecosystem function the following quantities were averaged in this way: the specific growth rate of the phytoplankton community, *μ*_*P*_, that of the implicitly represented zooplankton community, *μ*_*Z*_ (equation S-5, Supplementary online Methods), and the nutrient concentration, *N* (as a measure of nutrient drawdown). This was done for different values of the KTW and TD parameters, respectively, to examine the effects of different levels of diversity on the ecosystem response at different disturbance frequencies. As a control, the same model was run without either formulation for sustaining diversity, i.e., with the TD parameter, *ν* = 0, and the KTW parameter, *α* = 1. To examine the degree of complementarity^8^,^33^ the model results were compared to those for the single size (i.e., monoculture) having the greatest average productivity (specific growth rate), which was calculated numerically for each pulse frequency and period of interest (short- or long-term).

As a measure of size diversity, and hence of functional diversity, we calculate the continuous entropy for the assumed log-normal size distribution of phytoplankton^37^,


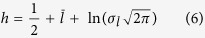


where 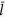 is the mean size, and 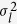 is the variance of the size distribution in log space. Note that although the original reference^37^ denoted this quantity as *μ*, here we refer to it as *h*, to avoid confusion with the specific growth rate defined above. For each pulse scenario, we quantify the degree of transgressive overyielding by *LR*_*trans*_^8^, which is the natural logarithm of the ratio of the specific growth rate of the diverse community to that of the single most productive size.

## Additional Information

**How to cite this article**: Smith, S. L. *et al*. Phytoplankton size-diversity mediates an emergent trade-off in ecosystem functioning for rare versus frequent disturbances. *Sci. Rep.* **6**, 34170; doi: 10.1038/srep34170 (2016).

## Supplementary Material

Supplementary Information

## Figures and Tables

**Figure 1:**
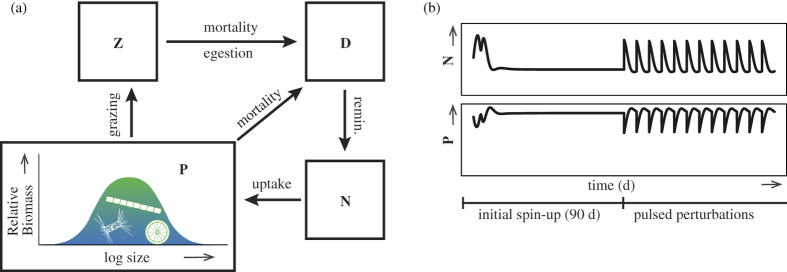
Diagram of the plankton ecosystem model, control version (**a**), which includes compartments (boxes) for nutrient, N, phytoplankton, P, zooplankton, Z, and detritus, D. The functional diversity of phytoplankton is represented by their size distribution (assumed log-normal) combined with size-scaled trait values. (**b**) Schematic of the setup for simulations, each of which includes an initial spin-up period of 90 d, followed by pulsed mortality events with corresponding nutrient addtions of differing frequency and intensity. For details see the Supplementary online Methods.
