# Supplementary material for: Phytoplankton size-diversity mediates an emergent trade-off in ecosystem functioning for rare versus frequent disturbances
Source: Sci Rep. 2016 Oct 17;6:34170. doi: 10.1038/srep34170 (PMC5066229; doi:10.1038/srep34170)
Supplement: Supplementary Information [file srep34170-s1.pdf]

Supplementary online Materials for: Phytoplankton size-diversity mediates an emergent trade-off in ecosystem functioning for rare versus frequent disturbances

S. Lan Smith[1,*], Sergio M. Vallina[2], and Agostino Merico[3,4]

[1] Ecosystem Dynamics Research Group, Research Centre for Global Change, JAMSTEC, Yokohama, Japan; e-mail: lanimal@jamstec.go.jp
[2] Institute of Marine Sciences (CSIC), 08003 Barcelona, Catalonia, Spain; e-mail: vallina@icm.csic.es
[3] Systems Ecology, Leibniz Center for Tropical Marine Ecology (ZMT), Bremen, Germany; e-mail: agostino.merico@leibniz-zmt.de
[4] Physics & Earth Sciences, Jacobs University, Bremen, Germany
[*] Corresponding author, S. Lan Smith
postal address: JAMSTEC, 3173-25 Showa-machi, Kanazawa-ku Yokohama, 236-0001 Japan
e-mail: lanimal@jamstec.go.jp
telephone: +81-45-778-5581, fax: +81-45-778-5706

Supplementary Methods

Continuous Size Distribution Model for Phytoplankton

We apply the 'adaptive dynamics' [Wirtz and Eckhardt, 1996, Norberg et al., 2001, Merico et al., 2009] approach to approximate the dynamics of the size distribution. Thus the equations for the rates of change of the mean and standard deviation of the distribution are written in terms of derivatives of the net growth rate, i.e., the 'fitness gradient' [Smith et al., 2011]. Here we also incorporate the inter-generational trait diffusion approach [Merico et al., 2014] as a means of sustaining diversity. Thus, the rates of change of the mean trait value and the trait variance are:

$$\frac{d\bar{l}}{dt} \approx \left\{ \sigma_l^2 \left[\frac{\partial}{\partial l} \left(\mu(N,l) - g(l) \right) + \nu \frac{\partial^3}{\partial l^3} \mu(N,l) \right] - 3\nu \frac{\partial}{\partial l} \mu(N,l) \right\} \Bigg|_{l=\bar{l}} \tag{S-1}$$

$$\frac{d\sigma_l^2}{dt} \approx \left\{ \sigma_l^2 \left[\sigma_l^2 \left(\frac{\partial^2}{\partial l^2}(\mu(N,l) - g(l)) + \nu \frac{\partial^4}{\partial l^4}\mu(N,l) \right) - 5\nu \frac{\partial^2}{\partial l^2}\mu(N,l) \right] + 2\nu\mu(N,l) \right\} \Bigg|_{l=\bar{l}} \tag{S-2}$$

where $\bar{l}$ is the mean of the distribution of size (l), σ_l^2 is its variance, $g(l)$ is the distribution of specific grazing rate over prey (phytoplankton) size from the continuous KTW formulation (derived below), and ν is the rate of trait diffusion [Merico et al., 2014]. The necessary derivatives with respect to size are provided below.

Total biomass and nutrient concentration

The community average growth rate of phytoplankton (over all size classes, l) can be approximated based on the assumed log-normal size distribution. Thus, the rate of change of the total biomass of the community, P_T, is approximated based on a Taylor expansion about the mean size, $\bar{l}$, assuming a Gaussian distribution [Merico et al., 2009]:

$$\frac{dP_T}{dt} \approx \frac{P_T}{2} \left\{ 2(\mu(N,l) - m_P) + \sigma_l^2 \left[\frac{\partial^2}{\partial l^2}\mu(N,l) + \nu \frac{\partial^4}{\partial l^4}\mu(N,l) \right] - 3\nu \frac{\partial^2}{\partial l^2}\mu(N,l) \right\} \Bigg|_{l=\bar{l}} - G_T \tag{S-3}$$

in which $G_T = g_{\max} Z Q(P_T)$ is the total grazing rate in terms of the feeding probability, Q, from equation (S-13). Here we have exploited the fact that the total grazing rate depends only on the total biomass, not on its distribution nor on the value of the prey switching parameter α [Vallina et al., 2014]. The second partial derivative of the specific growth rate (μ) will be negative at the mean value of log size, $\bar{l}$, assuming that the latter is near the optimal value of l, at which μ is maximal. That is, the community as a whole will grow more slowly than phytoplankton of precisely the mean (and optimal) size, because of the presence of other (sub-optimal) sizes.

The rate of change of the zooplankton biomass, Z, is:

$$\frac{dZ}{dt} = \mu_Z Z - m_Z Z^2 \tag{S-4}$$

where μ_Z is the specific growth rate of zooplankton (defined below), and m_Z is the mortality rate coefficient for zooplankton. The specific growth rate of zooplankton is:

$$\mu_Z = \beta_Z g_{\max} Q(P_T) \tag{S-5}$$

where β_Z is the assimilation efficiency of zooplankton, and $Q(P_T)$ is the feeding probability from equation (S-13).

The rate of change of the nutrient concentration, N, is:

$$\begin{aligned}\frac{dN}{dt} &= (1-\beta_Z)\,\epsilon_Z G_T + \Omega\left(m_P P_T + m_Z Z^2\right) \\ &- P_T\left\{\mu(N,l) + \frac{\sigma_l^2}{2}\left[\frac{\partial^2}{\partial l^2}\mu(N,l) + \nu\frac{\partial^4}{\partial l^4}\mu(N,l)\right] - \frac{3}{2}\nu\frac{\partial^2}{\partial l^2}\mu(N,l)\right\}\Bigg|_{l=\bar{l}}\end{aligned} \tag{S-6}$$

where fraction ϵ_Z of the un-assimilated grazing (first term) and fraction Ω of the zooplankton mortality (second term) are assumed to be remineralized instantaneously to N. The mass balance for detrital nitrogen, D, is:

$$\frac{dD}{dt} = (1-\beta_Z)\,(1-\epsilon_Z)\,G_T + (1-\Omega)\left(m_P P_T + m_Z Z^2\right) - k_D D \tag{S-7}$$

where k_D is the specific remineralization rate of detritus.

Size-scaled Kill-the-Winner grazing

The generalized grazing expression [Vallina et al., 2014], for the rate of grazing (by zooplankton) on discrete prey class i, having biomass P_i (mmol N m^{-3}), is:

$$G_i = g_{\max} Z \frac{\rho_i P_i^{\alpha}}{\sum_j^n \rho_j P_j^{\alpha}} \frac{P_T^{\beta}}{k_{sat}^{\beta} + P_T^{\beta}} \tag{S-8}$$

where $g_{\max}$ (d^{-1}) is the maximum grazing rate, Z (mmol N m^{-3}) is the biomass of (the implicit community of) zooplankton, ρ_i is the fixed preference for prey of discrete class i, parameter α determines the prey switching behavior, and parameters k_{sat} (mmol m^{-3}) and β determine the shape of the overall (total) grazing response in terms of total prey biomass, P_T (mmol N m^{-3}). The latter is the sum over all n prey classes of fixed prey preference times biomass:

$$P_T = \sum_j^n \rho_j P_j \tag{S-9}$$

Prey switching is determined by the ratio:

$$\delta_i(P_i) = \frac{\rho_i P_i^{\alpha}}{\sum_j^n \rho_j P_j^{\alpha}} \tag{S-10}$$

with $\alpha = 1$ giving 'passive' switching, resulting in competitive exclusion for prey, and $\alpha > 1$ giving active switching, resulting in kill-the-winner response [Vallina et al., 2014]. Dividing by P_i gives the specific loss rate of prey class i to grazing:

$$g_i = g_{\max} Z \frac{\rho_i P_i^{(\alpha-1)}}{\sum_j^n \rho_j P_j^{\alpha}} \frac{P_T^{\beta}}{k_{sat}^{\beta} + P_T^{\beta}} \tag{S-11}$$

This can be re-written in terms of the feeding probability Q, which depends on P_T, but not on P_i:

$$g_i = g_{\max} Z Q(P_T) \frac{\rho_i P_i^{(\alpha-1)}}{\sum_j^n \rho_j P_j^{\alpha}} \tag{S-12}$$

where

$$Q(P_T) = \frac{P_T^{\beta}}{k_{sat}^{\beta} + P_T^{\beta}} \tag{S-13}$$

For a continuous size distribution of prey, defined by probability density $P(l)$, the specific

loss to grazing for size l can be written:

$$g(l) = g_{\max} Z Q(P_T) \frac{\rho(l) P(l)^{(\alpha-1)}}{\int_{-\infty}^{\infty} \rho(l) P(l)^{\alpha} \, \mathrm{d}l} \tag{S-14}$$

where the total palatable prey is:

$$P_T = \int_{-\infty}^{\infty} \rho(l) P(l) \, \mathrm{d}l \tag{S-15}$$

with $\rho(l)$ defined as some continuous function of l. Here we assume $\rho(l) = 1$ for all l, so that the specific grazing rate simplifies to:

$$g(l) = g_{\max} Z Q(P_T) \frac{P(l)^{(\alpha-1)}}{\int_{-\infty}^{\infty} P(l)^{\alpha} \, \mathrm{d}l} \tag{S-16}$$

The size distribution of phytoplankton (prey) will be approximated as log-normal [Schartau et al., 2010, Wirtz, 2013] so that its probability density function, $P(l)$, in terms of log-size, l, is Gaussian:

$$P(l) = \frac{P_T}{\sigma_l \sqrt{2\pi}} e^{-\frac{1}{2}\left(\frac{l-\bar{l}}{\sigma_l}\right)^2} \tag{S-17}$$

where $\bar{l}$ is the (biomass weighted) mean log cell size and σ_l is the standard deviation of log cell size. Then, the normalizing integral in the denominator of equation (S-16) is:

$$\begin{aligned} \int_{-\infty}^{\infty} P(l)^{\alpha} \, \mathrm{d}l &= \int_{-\infty}^{\infty} \left[\frac{P_T}{\sigma_l \sqrt{2\pi}}\right]^{\alpha} e^{-\frac{\alpha}{2}\left(\frac{l-\bar{l}}{\sigma_l}\right)^2} \, \mathrm{d}l \\ &= \left[\frac{P_T}{\sigma_l \sqrt{2\pi}}\right]^{\alpha} \frac{\sigma_l \sqrt{2\pi}}{\sqrt{\alpha}} \end{aligned} \tag{S-18}$$

Substituting into equation (S-16) gives:

$$\begin{aligned} g(l) = g_{\max} Z Q(P_T) \left[\frac{\sigma_l \sqrt{2\pi}}{P_T}\right]^{\alpha} \frac{\sqrt{\alpha}}{\sigma_l \sqrt{2\pi}} \left[\frac{P_T}{\sigma_l \sqrt{2\pi}}\right]^{\alpha-1} e^{-\frac{\alpha-1}{2}\left(\frac{l-\bar{l}}{\sigma_l}\right)^2} \\ = \frac{g_{\max} Z Q(P_T)}{P_T} \sqrt{\alpha} \, e^{-\frac{\alpha-1}{2}\left(\frac{l-\bar{l}}{\sigma_l}\right)^2} \end{aligned} \tag{S-19}$$

Derivatives with respect to size

Derivatives of growth rate

The derivatives of the growth rate with respect to size (l) are needed to calculate the rates of change of the total biomass and the mean and variance of the size distribution. The first derivative of the specific growth rate, equation 5 (main text), with respect to l is:

$$\frac{\partial}{\partial l}\mu(N,l) = a_\mu \mu(N,l) - a_K \frac{K(l)}{\mu_{\max}(l)N}\left[\mu(N,l)\right]^2 \tag{S-20}$$

and its second derivative is:

$$\begin{aligned}\frac{\partial^2}{\partial l^2}\mu(N,l) = &\left[a_\mu - 2a_K\frac{K(l)\mu(N,l)}{N\mu_{\max}(l)}\right]\frac{\partial\mu(N,l)}{\partial l}\\ &- a_K(a_K - a_{mu})\frac{K(l)}{N\mu_{\max}(l)}\left[\mu(N,l)\right]^2\end{aligned} \tag{S-21}$$

Taking the derivative of each term of the above, in turn, gives the third derivative:

$$\begin{aligned}\frac{\partial^3}{\partial l^3}\mu(N,l) = &\left[a_\mu - 2a_K\frac{K(l)\mu(N,l)}{N\mu_{\max}(l)}\right]\frac{\partial^2\mu(N,l)}{\partial l^2}\\ &- 2a_K\frac{K(l)}{\mu_{\max}(l)N}\left[\frac{\partial\mu(N,l)}{\partial l}\right]^2\\ &- 2a_K(a_K - a_\mu)\frac{K(l)\mu(N,l)}{N\mu_{\max}(l)}\frac{\partial\mu(N,l)}{\partial l}\\ &- a_K(a_K - a_\mu)^2\frac{K(l)}{\mu_{\max}(l)N}\left[\mu(N,l)\right]^2\\ &- 2a_K(a_K - a_\mu)\frac{K(l)\mu(N,l)}{N\mu_{\max}(l)}\frac{\partial\mu(N,l)}{\partial l}\end{aligned} \tag{S-22}$$

and combining the third and fifth terms gives:

$$
\begin{aligned}
\frac{\partial^3}{\partial l^3}\mu(N,l) = &\left[a_\mu - 2a_K\frac{K(l)\mu(N,l)}{N\mu_{\max}(l)}\right]\frac{\partial^2\mu(N,l)}{\partial l^2} \\
&- 2a_K\frac{K(l)}{\mu_{\max}(l)N}\left[\frac{\partial\mu(N,l)}{\partial l}\right]^2 \\
&- 4a_K(a_K - a_\mu)\frac{K(l)\mu(N,l)}{N\mu_{\max}(l)}\frac{\partial\mu(N,l)}{\partial l} \\
&- a_K(a_K - a_\mu)^2\frac{K(l)}{\mu_{\max}(l)N}\left[\mu(N,l)\right]^2
\end{aligned} \tag{S-23}
$$

Again taking the derivative of each term, respectively, gives the fourth derivative of μ:

$$
\begin{aligned}
\frac{\partial^4}{\partial l^4}\mu(N,l) = &\left[a_\mu - 2a_K\frac{K(l)\mu(N,l)}{N\mu_{\max}(l)}\right]\frac{\partial^3\mu(N,l)}{\partial l^3} \\
&- 2a_K(a_K - a_\mu)\frac{K(l)\mu(N,l)}{N\mu_{\max}(l)}\frac{\partial^2\mu(N,l)}{\partial l^2} \\
&- 2a_K\frac{K(l)}{\mu_{\max}(l)N}\frac{\partial\mu(N,l)}{\partial l}\frac{\partial^2\mu(N,l)}{\partial l^2} \\
&- 2a_K(a_K - a_\mu)\frac{K(l)}{N\mu_{\max}(l)}\left[\frac{\partial\mu(N,l)}{\partial l}\right]^2 \\
&- 4a_K\frac{K(l)}{\mu_{\max}(l)N}\frac{\partial\mu(N,l)}{\partial l}\frac{\partial^2\mu(N,l)}{\partial l^2} \\
&- 4a_K(a_K - a_\mu)^2\frac{K(l)\mu(N,l)}{N\mu_{\max}(l)}\frac{\partial\mu(N,l)}{\partial l} \\
&- 4a_K(a_K - a_\mu)\frac{K(l)}{N\mu_{\max}(l)}\left[\frac{\partial\mu(N,l)}{\partial l}\right]^2 \\
&- 4a_K(a_K - a_\mu)\frac{K(l)\mu(N,l)}{N\mu_{\max}(l)}\frac{\partial^2\mu(N,l)}{\partial l^2} \\
&- a_K(a_K - a_\mu)^3\frac{K(l)}{N\mu_{\max}(l)}\left[\mu(N,l)\right]^2 \\
&- 2a_K(a_K - a_\mu)^2\frac{K(l)\mu(N,l)}{N\mu_{\max}(l)}\frac{\partial\mu(N,l)}{\partial l}
\end{aligned} \tag{S-24}
$$

which, after substituting equation (S-23) and collecting terms, simplifies to:

$$\frac{\partial^4}{\partial l^4}\mu(N,l) =$$

$$\left[\left(a_\mu - 2a_K\frac{K(l)\mu(N,l)}{N\mu_{\max}(l)}\right)^2 - 6a_K\frac{K(l)}{N\mu_{\max}(l)}\left(\frac{\partial\mu(N,l)}{\partial l} + (a_K - a_\mu)\,\mu(N,l)\right)\right]\frac{\partial^2\mu(N,l)}{\partial l^2}$$
$$-2a_K\frac{K(l)}{\mu_{\max}(l)N}\left[a_K\left(3 - 2\frac{K(l)\mu(N,l)}{N\mu_{\max}(l)}\right) - 2a_\mu\right]\left[\frac{\partial\mu(N,l)}{\partial l}\right]^2$$
$$-a_K^2(a_K - a_\mu)^2\frac{K(l)}{\mu_{\max}(l)N}\left[1 - 2\frac{K(l)\mu(N,l)}{N\mu_{\max}(l)}\right][\mu(N,l)]^2 \quad \text{(S-25)}$$

Derivatives of grazing rate

Here we take the derivatives with respect to l of the specific (to phytoplankton) grazing rate, equation (S-19), for use in equation (S-3).

The first derivative of the specific grazing rate with respect to l is:

$$\frac{\partial g(l)}{\partial l} = g_{\max}ZQ(P_T)\frac{1}{\int_{-\infty}^{\infty}P(l)^\alpha\,\mathrm{d}l}\frac{\partial P(l)^{(\alpha-1)}}{\partial l} \quad \text{(S-26)}$$

The derivative of $P(l)^{(\alpha-1)}$, based on equation (S-17), is then:

$$\frac{\partial P(l)^{(\alpha-1)}}{\partial l} = \frac{-(\alpha-1)}{\sigma_l^2}\left[\frac{P_T}{\sigma_l\sqrt{2\pi}}\right]^{(\alpha-1)}\left(l-\bar{l}\right)e^{-\frac{(\alpha-1)}{2}\left(\frac{l-\bar{l}}{\sigma_l}\right)^2} \quad \text{(S-27)}$$

Substituting equation (S-27) and the value of the normalizing integral, from equation (S-18), into equation (S-26) gives:

$$\frac{\partial g(l)}{\partial l} = -g_{\max}ZQ(P_T)\frac{(\alpha-1)}{\sigma_l^2}\left(l-\bar{l}\right)\frac{\left[\frac{P_T}{\sigma_l\sqrt{2\pi}}\right]^{\alpha-1}}{\left[\frac{P_T}{\sigma_l\sqrt{2\pi}}\right]^{\alpha}\frac{\sigma_l\sqrt{2\pi}}{\sqrt{\alpha}}}e^{-\frac{(\alpha-1)}{2}\left(\frac{l-\bar{l}}{\sigma_l}\right)^2}$$
$$= \frac{-(\alpha-1)}{\sigma_l^2}\left(l-\bar{l}\right)g(l) \quad \text{(S-28)}$$

At the mean size, $\bar{l}$, $\partial g(l)/\partial l = 0$, which based on equation (S-1) means that the grazing response will not directly cause changes in $\bar{l}$ (although indirect effects are possible through changes in

nutrient concentration).

Taking the derivative of equation (S-28) gives the second derivative:

$$\frac{\partial^2 g(l)}{\partial l^2} = \frac{-g_{\max} Z Q(P_T)}{P_T} \frac{(\alpha-1)\sqrt{\alpha}}{\sigma_l^2} e^{-\frac{(\alpha-1)}{2}\left(\frac{l-\bar{l}}{\sigma_l}\right)^2} + \frac{g_{\max} Z Q(P_T)}{P_T} \frac{(\alpha-1)^2\sqrt{\alpha}}{\sigma_l^4} \left(l-\bar{l}\right)^2 e^{-\frac{(\alpha-1)}{2}\left(\frac{l-\bar{l}}{\sigma_l}\right)^2} \tag{S-29}$$

which can be expressed in terms of $g(l)$ using equation (S-19):

$$\frac{\partial^2 g(l)}{\partial l^2} = \frac{-(\alpha-1)}{\sigma_l^2}\left[1 - \frac{(\alpha-1)(l-\bar{l})^2}{\sigma_l^2}\right] g(l) \tag{S-30}$$

For the second derivative evaluated at the mean size, as in equations (S-2) and (S-3), only the first term remains, and it can be expressed in terms of the specific grazing rate from equation (S-19):

$$\left.\frac{\partial^2 g(l)}{\partial l^2}\right|_{l=\bar{l}} = \frac{-(\alpha-1)}{\sigma_l^2} \left. g(l)\right|_{l=\bar{l}} \tag{S-31}$$

Table S1. Values of model parameters. Size-scaled parameters for phytoplankton and size-independent parameters for the implicit zooplankton community [Vallina et al., 2014], which exhibits passive prey switching for $\alpha = 1$ and active switching, i.e., 'Kill-the-Winner' response, for $\alpha > 1$.

Parameter	value	units	description
$\mu_{\max}(l)$	-	d^{-1}	size-scaled max. phytoplankton growth rate
$\mu^\star$	1.0	d^{-1}	ref. value of $\mu_{\max}$ at $l = 0$
a_μ	1.0	-	size-scaling factor for $\mu_{\max}$
$K(l)$	-	mmol N m^{-3}	size-scaled half-sat. value for phytoplankton
$K^\star$	0.1	mmol N m^{-3}	ref. value of K_N at $l = 0$
a_K	2.0	-	size-scaling factor for K_N
m_P	0.2	d^{-1}	rate coeff. for phytoplankton mortality
$g_{\max}$	1.0	d^{-1}	max. specific rate of grazing by zooplankton
k_{sat}	0.75	mmol N m^{-3}	half-sat. value for total grazing rate
ν	0.04048[a]	-	trait diffusion parameter
α	2.0[a]	-	parameter for prey switching
β	2	-	shape parameter for total feeding response
β_Z	0.4	-	net growth efficiency of zooplankton
ϵ_Z	0.33	-	remineralized fraction of un-assimilated grazing
Ω	0.25	-	remineralized fraction of all plankton mortality
m_Z	0.2	d^{-1} m^3 (mmol N)$^{-1}$	rate coeff. for zooplankton mortality
k_D	0.1	d^{-1}	specific remineralization rate of detritus

[a] default value used to generate Figs. 1 and 2.

References

A. Merico, J. Bruggeman, and K. Wirtz. A trait-based approach for downscaling complexity in plankton ecosystem models. *Ecol. Model.*, 220:3001–3010, doi: 10.1016/j.ecolmodel.2009.05.005, 2009.

A. Merico, G. Brandt, S. L. Smith, and M. Oliver. Sustaining diversity in trait-based models of phytoplankton communities. *Frontiers Ecol. Evol.*, 2:doi: 10.3389/fevo.2014.00059, 2014. doi: 10.3389/fevo.2014.00059.

J. Norberg, D. P. Swaney, J. Dushoff, J. Lin, R. Casagrandi, and S. A. Levin. Phenotypic diversity and ecosystem functioning in changing environments: A theoretical framework. *Proc. Natl. Acad. Sci.*, 98:11376–11381, 2001.

M. Schartau, M. R. Landry, and R. A. Armstrong. Density estimation of plankton size spectra: a reanalysis of ironex ii data. *J. Plankton Res.*, 31:1167–1184, doi:10.1093/plankt/fbq072, 2010.

S. L. Smith, M. Pahlow, A. Merico, and K. W. Wirtz. Optimality-based modeling of planktonic organisms. *Limnol. Oceanogr.*, 56:2080–2094, doi: 10.4319/lo.2011.56.6.2080, 2011.

S. M. Vallina, B. A. Ward, S. Dutkiewicz, and M. J. Follows. Maximal feeding with active prey switching: A kill-the-winner functional response and its effect on global diversity and biogeography. *Prog. Oceanogr.*, 120:doi: 10.1016/j.pocean.2013.08.001, 2014.

K. W. Wirtz and B. Eckhardt. Effective variables in ecosystem models with an application to phytoplankton succession. *Ecol. Model.*, 92:33–54, 1996.

Kai W. Wirtz. Mechanistic origins of variability in phytoplankton dynamics: Part i: niche formation revealed by a size-based model. *Marine Biology*, Feb 2013. doi: 10.1007/s00227-012-2163-7. URL http://dx.doi.org/10.1007/s00227-012-2163-7.

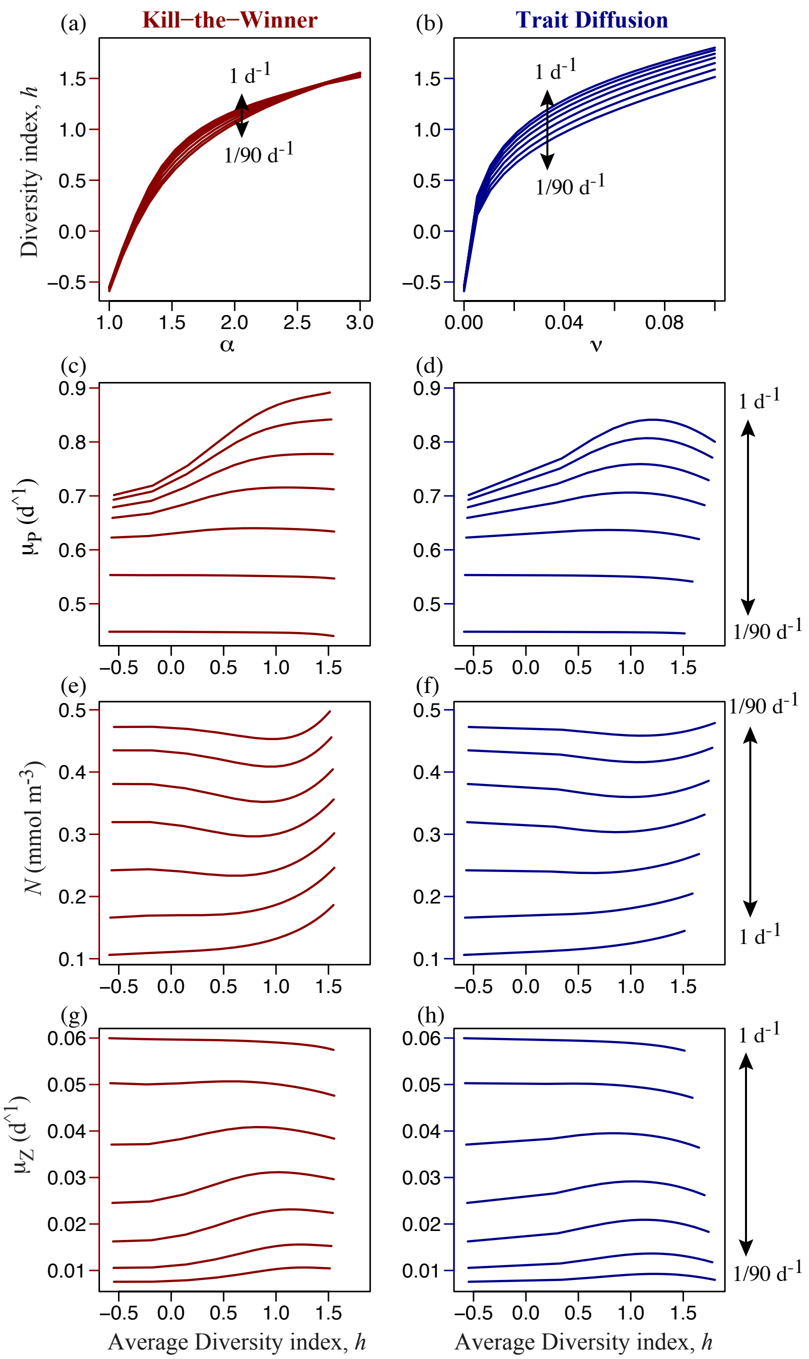

Figure S1: Size diversity index, h, averaged over 7 d following the first disturbance versus the a) KTW parameter α, and b) TD parameter ν. Vertical arrows specify frequencies of disturbance. Short-term Adaptive Capacity (AC) is quantified by avg. values over the same 7 d of: mean specific growth rate, μ_P, for the phytoplankton community (c, d), nutrient concentration, N (e, f), and specific growth rate of zooplankton, μ_Z (g, h), each plotted vs. h averaged over 7 d following the first disturbance.

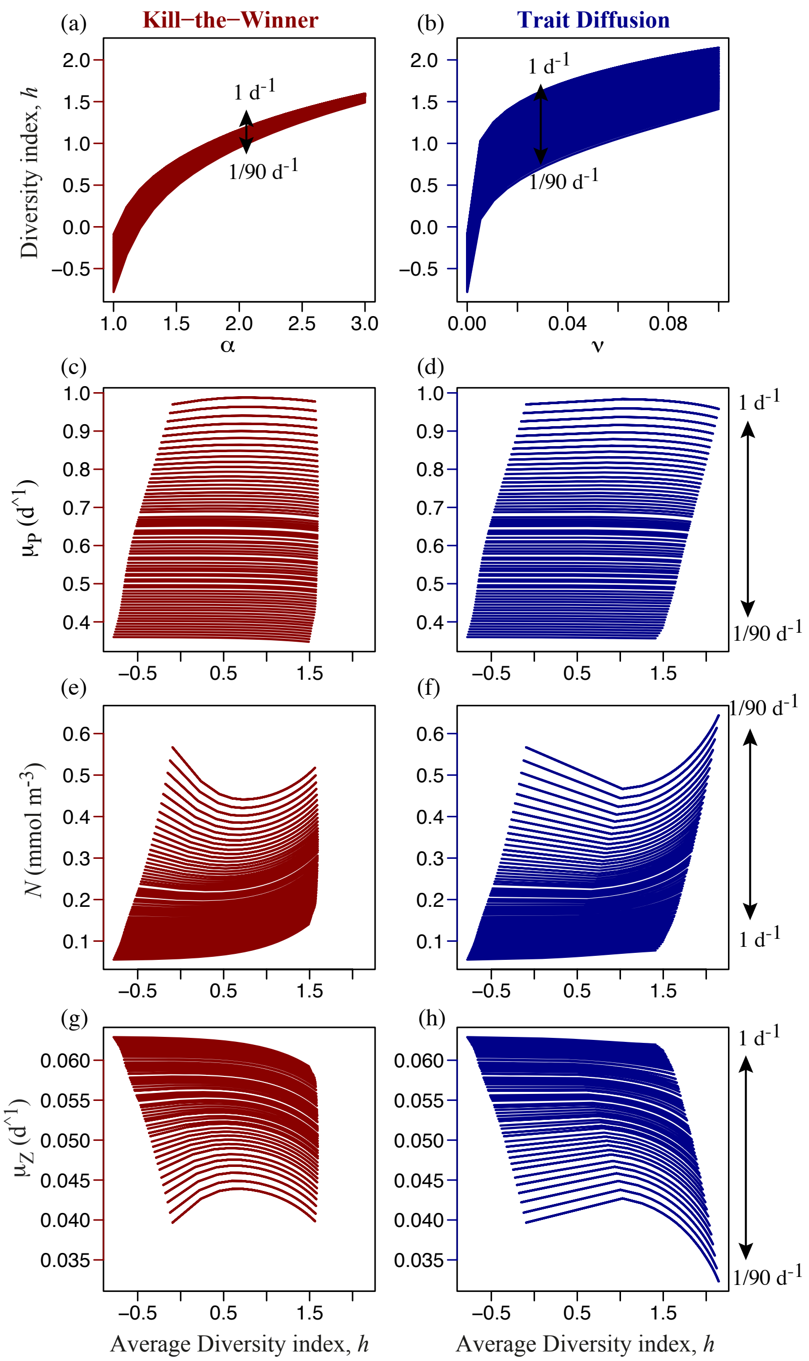

Figure S2: Size diversity index, h, averaged over 90 d following the first disturbance versus the a) KTW parameter α, and b) TD parameter ν. Vertical arrows specify frequencies of disturbance. Long-term Productivity (LP) is quantified by avg. values over the same 90 d of: mean specific growth rate, μ_P, for the phytoplankton community (c, d), nutrient concentration, N (e, f), and specific growth rate of zooplankton, μ_Z (g, h), each plotted vs. h averaged over 90 d following the first disturbance.
